# Tumour-suppressor microRNAs *let-7* and *mir-101* target the proto-oncogene *MYCN* and inhibit cell proliferation in *MYCN*-amplified neuroblastoma

**DOI:** 10.1038/bjc.2011.220

**Published:** 2011-06-07

**Authors:** J Buechner, E Tømte, B H Haug, J R Henriksen, C Løkke, T Flægstad, C Einvik

**Affiliations:** 1 Department of Paediatrics, University Hospital of North-Norway, 9038 Tromsø, Norway; 2 Paediatric Research Group, Department of Clinical Medicine, Faculty of Health Sciences, University of Tromsø, 9037 Tromsø, Norway

**Keywords:** neuroblastoma, N-myc, *MYCN*, *let-7*, *mir-101*, 3′UTR

## Abstract

**Background:**

MicroRNAs (miRNAs) regulate expression of many cancer-related genes through posttranscriptional repression of their mRNAs. In this study we investigate the proto-oncogene *MYCN* as a target for miRNA regulation.

**Methods:**

A luciferase reporter assay was used to investigate software-predicted miRNA target sites in the 3′-untranslated region (3′UTR) of *MYCN*. The miRNAs were overexpressed in cell lines by transfection of miRNA mimics or miRNA-expressing plasmids. Mutation of the target sites was used to validate *MYCN* 3′UTR as a direct target of several miRNAs. To measure miRNA-mediated suppression of endogenous N-myc protein, inhibition of proliferation and inhibition of clonogenic growth, miRNAs were overexpressed in a *MYCN*-amplified neuroblastoma cell line.

**Results:**

The results from this study show that *MYCN* is targeted by several miRNAs. In addition to the previously shown *mir-34a/c*, we experimentally validate *mir-449*, *mir-19a/b*, *mir-29a/b/c, mir-101* and *let-7e/mir-202* as direct *MYCN*-targeting miRNAs. These miRNAs were able to suppress endogenous N-myc protein in a *MYCN*-amplified neuroblastoma cell line. The *let-7e* and *mir-202* were strong negative regulators of *MYCN* expression. The *mir-101* and the let-7 family miRNAs *let-7e* and *mir-202* inhibited proliferation and clonogenic growth when overexpressed in Kelly cells.

**Conclusion:**

The tumour-suppressor miRNAs *let-7* and *mir-101* target *MYCN* and inhibit proliferation and clonogenic growth of *MYCN*-amplified neuroblastoma cells.

The transcription factor N-myc, which is encoded by the human *MYCN* proto-oncogene, belongs to the Myc family of DNA binding basic region/helix-loop-helix/leucine zipper (bHLHZip) proteins in which c-Myc, L-Myc and N-myc are the best characterised members (Meyer and Penn, 2008). The genomic sequences of *MYCN* and *c-MYC* share wide structural homology. Both genes consist of three exons, where the first exon is untranslated and exons 2 and 3 encode the translated regions (Kohl *et al*, 1986). N-myc and c-Myc proteins are of similar sizes (464 aa and 454 aa, respectively). However, the *MYCN* mRNA is longer, mainly because of a larger 3′-untranslated region (3′UTR). In addition to structural and sequence homologies within the Myc family, the functions of these proteins are closely related. Myc proteins heterodimerise with the bHLHZip-protein Max to a transcription factor complex that binds to specific E-box DNA motifs (5′-CACGTG-3′ or variants thereof) and activates transcription of genes involved in diverse cellular functions, including cell growth and proliferation, metabolism, apoptosis and differentiation (Bell *et al*, 2010; Larsson and Henriksson, 2010). In addition to Myc, Max also dimerises with the bHLHZip-proteins Mad/Mnt. These complexes also bind to E-box elements, but repress transcription through the recruitment of corepressors (Nilsson and Cleveland, 2003). The N-myc protein has recently been shown to repress *TrkA* and *p75NTR* expression by interaction with Sp1 and Miz-1 at proximal/core promoter regions. In this repression complex, N-myc recruited the histone deacetylase HDAC1 to silence gene expression by deacetylating chromatin at the promoter (Iraci *et al*, 2011). Similar transcriptional repression by N-myc has also been shown at an Sp1-binding site in the tissue transglutaminase (TG2) core promoter (Liu *et al*, 2007).

Given the fundamental role of Myc proteins on cellular processes, their activity in nontransformed cells needs to be spatially and timely controlled. Although *c-MYC* is expressed during all developmental stages and in a distinct pattern throughout the cell cycle of dividing cells (Hooker and Hurlin, 2006), *MYCN* expression is restricted mainly to the peripheral and central nervous system and epithelial cells during particular embryonal stages (Stanton *et al*, 1992). Expression is controlled at multiple levels, including gene transcription through upstream regulators, mRNA turnover, and protein activation or decay upon phosphorylation of specific protein residues (Meyer and Penn, 2008).

Dysregulation of Myc activity is an oncogenic hallmark in human malignancies. Activation of Myc is mainly caused by gene translocations or amplifications, or enhanced protein translation or stability, leading to overexpression of a structural normal protein (Vita and Henriksson, 2006; Albihn *et al*, 2010). Neuroblastoma is a common childhood solid tumour in which *MYCN* is amplified in ∼15% of cases. *MYCN* amplification (MNA) in the tumour is closely related to poor survival of the patients, despite all modern multi-modal treatment efforts (Maris *et al*, 2007; Maris, 2010). In contrast, non-amplified (non-MNA), low-stage neuroblastoma tumours have the propensity to differentiate into benign subtypes, or regress spontaneously. Paradoxically, these localised, low-risk tumours also have elevated N-myc activity. Their *MYCN* mRNA and protein levels exceed the levels of high-risk non-MNA tumours with poor outcome, but do not reach those of MNA tumours (Cohn *et al*, 2000; Tang *et al*, 2006; Westermann *et al*, 2008). It has therefore been supposed that neuroblastoma cells with moderately elevated N-myc sustain the capacity to undergo apoptosis and neuronal differentiation (Edsjo *et al*, 2004).

Despite its clinical significance, it is still largely unknown how *MYCN* expression is regulated in neuroblastoma. Here, we have investigated how microRNAs (miRNAs) contribute to the control of *MYCN* expression in MNA neuroblastoma cells. MiRNAs are a class of small (19–22 nt), non-coding RNA molecules that repress protein expression through imperfect binding to sequences in the 3′UTR of target mRNAs. Most miRNAs are transcribed as long monocistronic, bicistronic or polycistronic primary transcription units (pri-miRNAs) by RNA polymerase II, and cleaved by a series of cellular processing events to produce mature miRNAs (Siomi and Siomi, 2009). The degree of complementarity between mature miRNAs and target mRNAs determines the mechanism responsible for blocking protein synthesis. In mammals, miRNA–mRNA interactions are most often through imperfect base pairing, resulting in translational repression. It has been estimated that 30% of all human genes are regulated by miRNAs (Lewis *et al*, 2005).

In the recent years, several studies have been carried out to investigate how N-myc as a transcription factor affects expression of miRNAs (Stallings *et al*, 2010). On the contrary, only little is known about *MYCN* as a miRNA target. The tumour-suppressor miRNA *mir-34a* has been experimentally validated to directly target the 3′UTR sequence of *MYCN* (Wei *et al*, 2008). In addition, a miRNA binding site for *mir-101* has also been reported in the *MYCN-*3′UTR sequence (Lewis *et al*, 2005). However, a more systematic screening for miRNA binding sites and validation studies of miRNA/*MYCN-*3′UTR interactions have to our knowledge not been performed. It is well established that cellular proto-oncogenes are regulated by miRNAs, and that disturbances in these relations contribute to cancer development.

In this study, we have investigated the 3′UTR of the *MYCN* proto-oncogene for conserved miRNA binding sites. We established several miRNAs as *MYCN*-controlling miRNAs, and define a subset with antiproliferative and anticlonogenic properties.

## Materials and methods

### Cell lines

The MNA neuroblastoma cell lines SMS-KCN, SMS-KCNR, SMS-KANR, SK-N-BE(2), Kelly and the non-MNA cell line SK-N-AS were grown in RPMI-1640 medium, MNA IMR-32 and LAN5 in DMEM medium with 1% NEAA and 2 mM glutamine, and non-MNA SH-SY-5Y in HAM-F12 with 1% NEAA and 2 mM glutamine. The embryonic kidney cell line HEK293 was grown in DMEM. All media were supplemented with 10% FBS. Cells were maintained in a humidified 37 °C incubator with 5% CO_2_, supplied with fresh complete medium every 3 days, and sub-cultured before confluence was reached.

### Genomic DNA from patient samples and neuroblastoma cell lines

Genomic DNA samples from 39 primary neuroblastoma tumours (34 MNA and 5 non-MNA) were kindly provided by Professor T Martinsson (Gothenborg University, Sweden) after informed consent from the patients and ethical approval by the institution. Genomic DNA from neuroblastoma cell lines was isolated with the DNeasy Blood and Tissue kit (Qiagen, Crawley, UK) according to the manufacturer's protocol.

### Screening for mutations in the *MYCN* 3′UTR

The *MYCN* 3′UTR in each DNA sample was amplified in a 50 *μ*l PCR reaction, using 25 ng genomic DNA, Platinum Taq polymerase (Invitrogen, Carlsbad, CA, USA) and 10 nM primers (Supplementary Table 1). Bi-directional sequencing was performed in 10 *μ*l reactions, using 50 ng of purified *MYCN* 3′UTR, BigDye3.1 reagent (Applied Biosystems, Carlsbad, CA, USA) and 10 nM forward or reverse primer. Sequences were analysed on an ABI Prism (Applied Biosystems) using the in-house Sequencing Core Facility.

### Exogenously overexpression of miRNAs

The sequences for *pre-mir-34a*, *-34c* and *-106b* flanked by 250 nt genomic sequence in both directions were amplified from SK-N-BE(2) genomic DNA using Platinum Taq polymerase (Invitrogen) and primers as described in Supplementary Table 1. *Pre-mir-449a* and *-449b*, which are expressed from the mir-449 cluster, were amplified from SK-N-BE(2) DNA as a bi-cistronic mir-449a/449b sequence (‘mir-449ab’). The PCR products were cloned into the MCS of the expression vector pcDNA6.2-EmGFP (Invitrogen) by In-Fusion cloning (Clontech Laboratories, Mountain View, CA, USA) according to the manufacturer's recommendations. Bi-directional DNA sequencing verified all vectors. All other miRNAs used in this study were purchased as miRNA mimics (Shanghai GenePharma, Shanghai, China) as listed in Supplementary Table 1. Overexpression of miRNAs was confirmed by miRNA-specific RT–qPCR as described (Buechner *et al*, 2011).

As negative controls, we used pcDNA6.2-EmGFP containing *pre-mir-346* in vector-based miRNA experiments and the Negative Control mimic (Shanghai GenePharma) for mimic-based expression.

### Luciferase/MYCN-3′UTR expression constructs

To generate the pMIR-MYCN-UTR vector, the full-length MYCN-3′UTR sequence was amplified from genomic DNA and cloned into the MCS of the *Firefly* luciferase expressing pMIR-REPORT (Ambion, Austin, TX, USA). QuikChange Multi Site-directed Mutagenesis Kit (Stratagene, La Jolla, CA, USA) was used to specifically mutate individual miRNA seed sequences. To disrupt miRNA binding, a two-base mismatch within position 2–6 of the corresponding *MYCN-*3′UTR seed sequence was introduced (Doench and Sharp, 2004) (Supplementary Figure 1). To ensure complete disruption of miRNA seed sequences in selected non-rescued cases, we extended the mutations to include a complete seed mismatch (position 2–8). Mutagenesis was performed according to the Stratagene's standard protocol. All mutagenesis primers are listed in Supplementary Table 1. All mutations were confirmed by bi-directional sequencing.

### Luciferase reporter assay (LRA)

LRAs were performed as previously described (Henriksen *et al*, 2011). Briefly, HEK293 cells were co-transfected with 20 ng pGL4.75[hRluc/CMV] (Promega, Madison, WI, USA), 2900 ng miRNA expression vector or 0.43 *μ*l miRNA mimic (20 *μ*M), and 100 ng of either wild-type pMIR-MYCN-UTR or a mutant variant. At 48 h hours after transfection, *Renilla* and *Firefly* luciferase activities were analysed using the Dual Luciferase Assay (Promega). Each miRNA transfection was done in triplets and independently repeated at least three times, resulting in at least nine LRAs for each individual miRNA. Luciferase activities were analysed in duplicates. Normalisation included two steps: first, the *Firefly* luciferase activity was normalised to the *Renilla* luciferase activity, and second, the normalised luciferase activity of transfected NC (pre-mir-346 or Negative Control mimic) was set as relative luciferase activity of 1. The PASW Statistics 18 software (SPSS Inc., Chicago, IL, USA) was used for data analyses and boxplot charts.

### Western blotting

Cells were transfected in six-well plates with 5 *μ*l miRNA mimic (20 *μ*M) using a standard Lipofectamine2000 reverse transfection protocol (Invitrogen). After 48 h, protein expression was determined by western blotting as previously described (Henriksen *et al*, 2011) with primary antibodies against N-myc (1 : 500, Calbiochem/Merck, Darmstadt, Germany), *β*-Actin (1 : 1250, Sigma-Aldrich Corp., St Louis, MO, USA), and the secondary antibodies IRDye800CW (1 : 5000, Rockland, Gilbertsville, PA, USA) and Alexa Fluor 680 (1 : 5000, Invitrogen).

### Cell proliferation assay

Cell growth was monitored continuously in 16-well E-plates on the xCELLigence system (Roche, Mannheim, Germany). Kelly cells were seeded in 160 *μ*l media (15 000 cells per well) and transfected in triplicates 4–6 h later with 60 *μ*l of a transfection mix containing 0.2 *μ*l Lipofectamine2000 and 0.6 *μ*l miRNA mimic (20 *μ*M). Proliferation was recorded automatically as cell index every 30 min for a minimum of 72 h. The cell index is derived from changes in electrical impedance as the cells interact with interdigitated microelectrodes integrated on the bottom of the E-plate.

### Clonogenic cell assay

To investigate the capability of single cells to survive and proliferate after transfection of specific miRNA mimics, cells were transfected as described and seeded in six-well-dishes in a density of 200 cells per well. Staining was performed as previously described (Henriksen *et al*, 2011).

## Results

### Mutational screening of the *MYCN* 3′UTR

**Figure 1:**
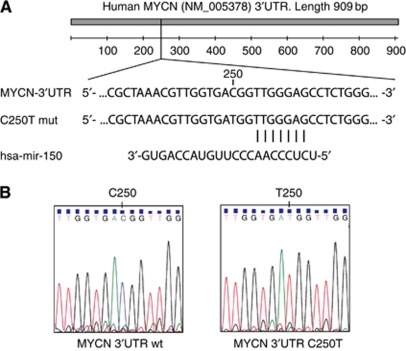
C to T variation at position 250 in the MYCN 3′UTR. Mutational analysis of the human MYCN 3′UTR (NM_005378). (**A**) Schematic overview of the MYCN 3′UTR including the rs922 SNP (C250T mut) located 3 bp upstream of the predicted target sequence for mir-150. (**B**) Exclusively homozygous C/C or T/T genotype variants were detected in neuroblastoma cell lines and primary tumours.

In order to reveal mutations or single-nucleotide polymorphisms (SNPs) that could potentially disturb miRNA-mediated suppression of *MYCN* expression in neuroblastoma, the entire *MYCN-*3′UTR sequence from 7 MNA neuroblastoma cell lines (SMS-KCN, SMS-KCNR, SMS-KANR, SK-N-BE(2), Kelly, IMR-32 and LAN5) and 39 neuroblastoma primary tumours (34 MNA and 5 non-MNA) was sequenced. According to the human *MYCN* germ-line sequence (Genbank accession no. NM_005378) six cell lines contained the wild-type *MYCN-*3′UTR sequences, whereas the LAN5 cell line was found to carry a homozygous point mutation at position 250, changing a cytosine to a thymine (C250T) (Figures 1A and B). The identical homozygous mutation was found in 35% of MNA primary tumours (12 of 34) and 20% of non-MNA primary tumours (1 of 5). The C/T variation at this genomic position is termed rs922 in the NCBI RefSNP database. We were not able to detect heterozygous variants at this position, or any other mutations in the *MYCN-*3′UTR sequence.

### Prediction of miRNAs targeting the *MYCN* 3′UTR

**Figure 2:**
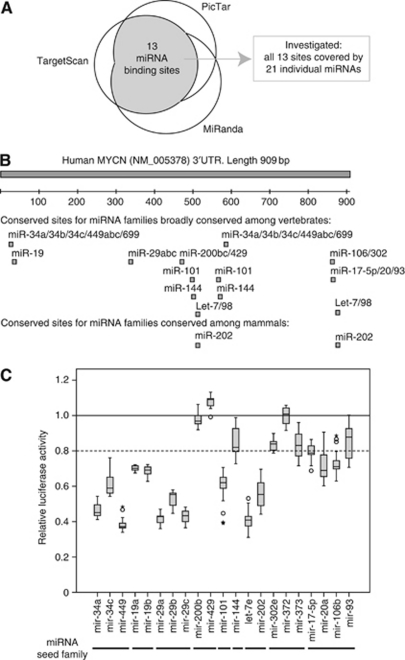
Prediction and experimental investigation of *MYCN*-targeting miRNAs. (**A**) Intersection of three web-based miRNA target prediction programmes (TargetScan, PicTar and miRanda) predicted 13 conserved binding sites for miRNAs broadly conserved among vertebrates. A total of 21 individual miRNAs were selected for target site validation. (**B**) Schematic overview of *MYCN* 3′UTR showing the localisation of the predicted miRNA target sites investigated in luciferase reporter assays (LRAs). (**C**) Results from LRAs shown as boxplot diagrams. Horizontal bars indicate miRNAs predicted to target identical target sites (miRNA seed families). *Firefly* luciferase was normalised to *Renilla* luciferase activity, and then normalised to the median activity of the control miRNA. Each box represents the distribution of the activity measured for a single miRNA (at least 9 individual transfections, range 9–36). Ends of boxes define the 25th and 75th percentile, a line defines the median, and bars define the lowest and highest value except outliers and extreme values, which are indicated by circles and asterisks, respectively. Reduction in relative luciferase activity of ⩾20% was defined to be indicative for a miRNA/*MYCN-*3′UTR interaction.

As a first step to identify *MYCN*-targeting miRNAs, we combined three miRNA target prediction programmes, TargetScan 5.1 (Lewis *et al*, 2003), MiRanda (John *et al*, 2004) and PicTar (Lall *et al*, 2006), to predict potential miRNA binding sites in the full-length *MYCN-*3′UTR sequence. TargetScan, which uses site and miRNA conservation across different species as selection criteria, predicted 43 individual miRNAs with broad conservation among vertebrates, targeting a total of 14 different conserved miRNA binding sites. We intersected the TargetScan miRNA seed subset with the predictions from the MiRanda and PicTar programmes. Of the 14 conserved binding sites predicted by TargetScan, 13 were also predicted by one of the other programmes (Figure 2A). To completely cover these 13 binding sites, we selected 20 individual miRNAs for experimental validation (Supplementary Table 2). We finally expanded the panel to include *mir-202*, a *let-7* miRNA family member conserved only among mammals (Figure 2B).

### Experimental validation of *MYCN-*3′UTR target sites by LRAs

We used LRA to experimentally validate whether the 21 candidate miRNAs were able to target the *MYCN-*3′UTR sequence. Briefly, the full-length *MYCN* 3′UTR (909 bp) was cloned into the pMIR-Report vector downstream of the luciferase gene, generating pMIR-MYCN-UTR. This vector was co-transfected with either miRNA mimics or miRNA-expression plasmids into HEK293 cells, and luciferase activity was measured and compared with co-transfections with a negative control miRNA. Overexpression of miRNAs was confirmed by qRT–PCR assays (data not shown). As shown in Figure 2C, 14 individual miRNAs (representing 9 of the 13 predicted miRNA binding sites) reduced the normalised luciferase activity by >20%. These miRNAs belong to six distinct miRNA seed families: the *mir-34abc/449abc/699, mir-19a/b, mir-29abc, mir-101, let-7/98/202,* and *mir-17/20/93/106/519* family.

**Figure 3:**
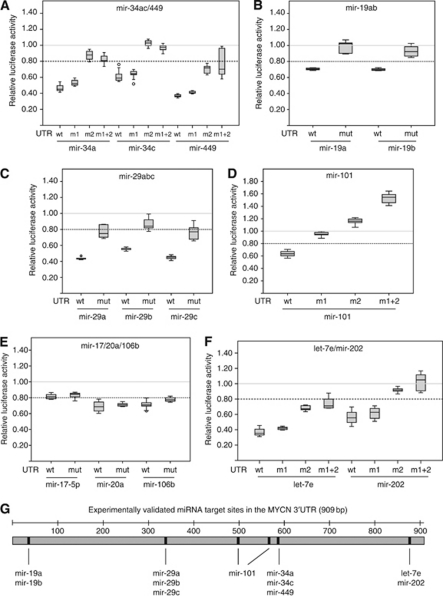
Experimental validation of miRNA target sites in the 3′UTR sequence of MYCN. The predicted target sites for the miRNA families *mir-34a/-c/-449* (**A**), *mir-19a/-b* (**B**), *mir-29a/-b/-c* (**C**), *mir-101* (**D**), *mir-17/-20a/-106b* (**E**) and *let-7/mir-202* (**F**) were mutated using site-directed mutagenesis. wt=wild-type 3′UTR sequence; mut=3′UTR sequences mutated in the seed sequence corresponding to the investigated miRNA (Supplementary Figure 1). For miRNA families containing two potential target sites, ‘m1’ and ‘m2’ denote mutations of the 5′-most and 3′-most target site, respectively. ‘m1+2’ means that both target sites are mutated. The target site mutants were investigated in LRAs and compared with the wild-type *MYCN-*3′UTR sequence. Graphical presentations are similar to that described for Figure 2. (**G**) Overview of the six experimentally confirmed miRNA target sites in the *MYCN* 3′UTR. The individual miRNAs used in the validation experiments are shown.

To investigate if the observed reduction in luciferase activity was caused by direct miRNA/*MYCN*-3′UTR interactions, we introduced point mutations into the seed sequences of the nine potential miRNA binding sites in the pMIR-MYCN-UTR vector to abolish miRNA binding (Supplementary Figure 1). For miRNAs with two potential binding sites, both seeds were mutated individually (‘m1’ and ‘m2’, where ‘m1’ is the 5′-most binding site) as well as combined (‘m1+2’). We then performed LRAs with the 14 luciferase-repressing miRNAs, and compared the luciferase activities from the respective UTR-mutated and wild-type reporters. As shown in Figure 3, a rescue in luciferase activity was observed for several mutated target sites, indicating specific miRNA/*MYCN*-3′UTR interactions. Specifically, *mir-34a/-34c/-449* (Figure 3A), *mir-19a/-19b* (Figure 3B), *mir-29a/-29b/-29c* (Figure 3C), *mir-101* (Figure 3D) and *let-7e/mir-202* (Figure 3F) were found to target the *MYCN*-3′UTR sequence at positions 581–587 (seed 2), 32–38, 334–340, 494–500 and 563–569 (seeds 1 and 2) and 870–876 (seed 2), respectively. We were not able to confirm specific targeting of *mir-17/-20a/-106b* to the predicted target site at position 859–865 (Figure 3E). An alternative binding site for *mir-17/-20a/-106b* at position 685–690 was uniquely predicted by the MiRanda software. However, mutation of this target site did not substantially affect the luciferase activity (Supplementary Figure 2a).

To exclude the possibility that the lack of luciferase rescue was because of incomplete destruction of target sites, we extended the mismatch from 2 to 7 nt (complete seed mismatch) on selected target sites (*mir-17/-20a/-106b* and *mir-34a/-34c/-449* – Supplementary Figure 1). However, the luciferase activities remained unaffected (Supplementary Figures 2b and c).

In summary, our data reveal six conserved miRNA binding sites in the 3′UTR structure of *MYCN* (Figure 3G). We were able to experimentally validate *mir-34a*, *-34c*, *-449*, *-19a*, *-19b*, *-29a, -29b, -29c, -101, -202* and *let-7e* as *MYCN*-targeting miRNAs.

### The rs922 SNP does not affect miRNA binding

The *mir-150* is the only miRNA predicted to bind in close proximity to the rs922 SNP (Figure 1A, Supplementary Table 2). Overexpression of *mir-150* in the LRA did not reduce luciferase expression from pMIR-MYCN-UTR or a mutated luciferase reporter containing the rs922 SNP (Supplementary Figure 3a). We then investigated whether the confirmed *MYCN*-targeting miRNAs were influenced by the rs922 SNP. We did not observe substantial luciferase rescue for the validated *MYCN*-targeting miRNAs when the rs922-containing luciferase reporter was compared with the 3′UTR wild-type sequence (Supplementary Figure 3a). This indicates that the rs922 SNP does not significantly influence miRNA-mediated suppression of *MYCN* expression.

### MiRNA-mediated *MYCN* suppression in MNA Kelly cells

As the tumour-suppressor function of *mir-34a* and *-34c* in MNA neuroblastoma is well documented (Welch *et al*, 2007; Cole *et al*, 2008; Wei *et al*, 2008; Tivnan *et al*, 2011), we selected the remaining eight experimentally validated *MYCN*-targeting miRNAs (mir*-19a*, *-19b*, *-29a, -29b, -29c, -101, -202* and *let-7e*) for further functional analyses.

**Figure 4:**
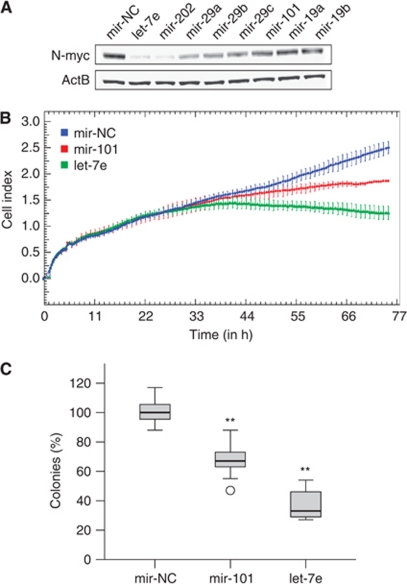
The miRNA-mediated N-Myc downregulation and growth inhibition in MNA Kelly cells. (**A**) A representative western blot showing strong N-Myc protein reduction upon transfection of *let-7e*, *mir-202*, *mir-29a/-b/-c* and *mir-101*. *Mir-19a/-b* reduced N-Myc levels only slightly. (**B**) The 72-h continuous monitoring of cell proliferation after transfection of *let-7e*, *mir-101* or a negative control (mir-NC) mimic into Kelly cells. (**C**) Clonogenic growth of Kelly cells was significantly impaired after transfection of *let-7e* and *mir-101* mimics, compared with the negative control (^**^P<0.001, Student's *t*-test). Each boxplot represents the distribution of colony numbers from three independent transfections, normalised to the median number of the negative control.

To investigate the capability of the selected miRNAs to downregulate endogenous N-myc protein in MNA neuroblastoma, we performed transient miRNA overexpression experiments and analysed N-myc expression by western blotting. As shown in Figure 4A, all eight miRNAs reduced endogenous N-myc protein expression in Kelly cells. Particularly, *let-7e* and *mir-202* were strong negative regulators of *MYCN* expression.

### *Let-7e* and *mir-101* inhibit cell proliferation and clonogenic growth of MNA Kelly cells

To investigate the functional role of *MYCN*-targeting miRNAs in MNA neuroblastoma, we continuously monitored the proliferation of Kelly cells after transfection with miRNA mimics. As shown in Figure 4B, proliferation was significantly impaired when cells were transfected with *mir-101* and *let-7e*. *Mir-202*, which targets the same seed sequence as *let-7e*, suppressed proliferation of Kelly cells similar to that observed for *let-7e* (Supplementary Figure 4). Cell proliferation was not substantially altered when *mir-19a/b* or *mir-29a/b/c* were overexpressed in Kelly cells (data not shown).

We further investigated the long-term effect of transient *let-7e* and *mir-101* overexpression on clonogenic cell growth. Kelly cells were transfected with *let-7e* and *mir-101* miRNA mimics and plated at 200 cells per well in six-well tissue culture plates. Visible cell clones were counted after 2 weeks of incubation. Compared with the negative control mimic, overexpression of *let-7e* and *mir-101* in Kelly cells reduced colony formation by 80% and 50%, respectively (Figure 4C).

In conclusion, we established that the let-7 family miRNAs, *let-7e* and *mir-202*, and *mir-101* have strong antiproliferative properties in the MNA neuroblastoma cell line Kelly.

## Discussion

Deregulated *MYCN* expression is a hallmark in high-risk neuroblastoma. Here, we aimed to investigate how miRNAs contribute to *MYCN* regulation. We systematically investigated the *MYCN-*3′UTR sequence for potential miRNA binding sites. We used LRAs to show that the 3′UTR sequence is directly targeted by several miRNAs (*mir-34a*, *-34c*, *-449*, *-19a*, *-19b*, *-29a, -29b, -29c, -101, -202* and *let-7e*). These miRNAs were further shown to decrease N-myc protein expression when overexpressed in the MNA neuroblastoma cell line Kelly. Finally, we showed that *let-7e*, *mir-101* and *mir-202* efficiently inhibit proliferation and clonogenic cell growth in Kelly cells.

It has been reported that certain mutations and SNPs in the 3′UTR of cancer-related genes increase cancer susceptibility and may allow the cancer cell to escape miRNA regulation (Pelletier and Weidhaas, 2010). Here, we show that mutations in the *MYCN* 3′UTR are rare, both in MNA and non-MNA neuroblastoma cells. We detected a single SNP (rs922) at position 250 (C250T) of the 3′UTR, which did not impair miRNA binding. We were unable to detect heterozygous C/T variants in our samples, which might be explained by the fact that only one allele is amplified in MNA samples, and by the limited number of investigated non-MNA samples.

*The mir-19a* and *mir-19b*, which are both expressed from the oncogenic mir-17-92 cluster, were validated to target the *MYCN* 3′UTR in the luciferase reporter assay system in HEK293 cells. However, we were not able to demonstrate a significant N-Myc protein downregulation after overexpression in Kelly cells. As *MYCN* itself is a direct activator of the mir-17-92 cluster (Loven *et al*, 2010), we assume that the *MYCN*-targeting function of *mir-19a/-b* is disturbed in MNA neuroblastoma cells.

The mir-34 family consists of *mir-34a*, *-34b* and *-34c*. The *mir-34a* is encoded by a locus at chromosome 1p36, whereas *mir-34b* and *-34c* are coexpressed from a locus at chromosome 11q23. Both regions are commonly hemizygously deleted in neuroblastoma (Attiyeh *et al*, 2005). In contrast to *mir-34b*, both *mir-34a* and *mir-34c* have been shown to inhibit cell growth in several neuroblastoma cell lines with 1p36 hemizygous deletion (Welch *et al*, 2007; Cole *et al*, 2008; Wei *et al*, 2008). The *mir-34a* has several experimentally validated targets involved in cellular proliferation, including *MYCN* (Hermeking, 2010). In this study, we confirm that *mir-34a* directly targets the 3′UTR sequence of *MYCN.* Whereas Wei *et al* (2008) concluded that both *mir-34* target sites were required to obtain maximum *MYCN* suppression by *mir-34a*, we found the 3′-most target site (position 581–587) alone to be responsible for most of the suppressive effect. Not even complete destruction of the 5′-most target site (position 23–29) could significantly rescue luciferase activity to indicate that this site is involved in *mir-34a*-mediated repression of *MYCN* (Supplementary Figure 2). This result is supported by data from a study performed by Stallings and co-workers (Welch *et al*, 2007), who were unable to show *mir-34a-*mediated suppression of a luciferase reporter containing only the 5′-most *mir-34* target site. The observed discrepancy could be explained by the different 3′UTR sequences and cells used in the LRAs. Whereas we used the full-length 909 nt *MYCN-*3′UTR sequence and HEK293 cells, Wei *et al* (2008) used a longer sequence including additional 418 nt of the *MYCN* coding sequence and SK-N-AS cells. We also verified that *mir-34c* and *mir-449*, but not *mir-34b*, target the *MYCN* 3′UTR similar to *mir-34a* (Figures 2C and 3A, Supplementary Figure 2 and data not shown).

In a study to validate the TargetScan algorithm, *mir-101* was predicted to potentially target two sequences in the *MYCN* 3′UTR (Lewis *et al*, 2003). The 5′-most target site (position 495–500) was mutated in a 409 nt 3′UTR fragment coupled to a luciferase reporter to verify it as a target of endogenous *mir-101* in HeLa cells. Here, we confirm *mir-101* as a *MYCN*-regulating miRNA able to suppress *MYCN* expression from both predicted target sites (Figures 2C and 3D). In addition, we show that *mir-101* inhibits proliferation of MNA Kelly cells. These data from MNA neuroblastoma confirm and extend previous reports showing tumour-suppressor properties of *mir-101* in different other cancer types (Varambally *et al*, 2008; Strillacci *et al*, 2009; Su *et al*, 2009; Cao *et al*, 2010; Wang *et al*, 2010; Zhang *et al*, 2011).

Xu *et al* (2009) have previously shown that *mir-29* directly regulates B7-H3, a surface glycoprotein of the B7/CD28 family that is expressed on a wide variety of solid tumour cells, including neuroblastoma. B7-H3 has immunoinhibitory effects protecting neuroblastoma cells from NK-mediated cytotoxicity (Castriconi *et al*, 2004). In addition, B7-H3 is the target of the monoclonal antibody 8H9 (Xu *et al*, 2009) that showed promising results when used in compartmental radioimmunotherapy (cRIT) in a clinical trial for CNS-relapsed high-risk neuroblastoma (Kramer *et al*, 2010). Compared with normal tissue, *mir-29* was found significantly lower expressed in neuroblastoma cells, contributing to a higher expression of B7-H3 on neuroblastoma cell surfaces (Xu *et al*, 2009). It has been suggested that restoration of *mir-29* and subsequent translational inhibition of B7-H3 might therefore prove therapeutically beneficial, both by sensitising neuroblastoma cells to NK/T-cell-mediated immunotoxicity and by protecting B7-H3 expressing normal tissue from 8H9-related toxicity (Xu *et al*, 2009). Our data extend the therapeutical potential of *mir-29* as it was shown to directly target *MYCN*.

The human let-7 miRNA family consists of 10 different mature *let-7* sequences that are derived from 13 precursors (Roush and Slack, 2008). Overexpression of *let-7* has been shown to inhibit proliferation of breast cancer (Zhao *et al*, 2011), lung cancer (Johnson *et al*, 2007), prostate cancer (Dong *et al*, 2010), colon cancer (Akao *et al*, 2006), malignant melanoma (Schultz *et al*, 2008) and glioblastoma (Lee *et al*, 2011) cell lines. Several important cell cycle regulators, including cyclins, cyclin-dependent kinases (CDKs), Ras, HMGA2 and c-Myc have previously been confirmed to be targets of *let-*7 (Johnson *et al*, 2005, 2007; Lee and Dutta, 2007; Schultz *et al*, 2008; Kim *et al*, 2009). We have now added the *MYCN* oncogene to the list of cell cycle regulators targeted by *let-7*. The observed growth-inhibitory effect of *let-7e* on MNA neuroblastoma cells is most likely because of the combined suppression of several *let-7* targets involved in cell proliferation.

In summary, we were able to define a subset of miRNA that are able to regulate *MYCN* expression when overexpressed in MNA neuroblastoma cells. To what extent the N-myc protein is regulated by endogenous levels of these miRNAs, and if altered levels contribute to neuroblastoma development, needs to be addressed in further studies. Recent data from miRNA profiling studies show that *let-7e*, *mir-29a* and *mir-29c* are significantly lower expressed in MNA primary tumours compared with non-MNA tumours (Schulte *et al*, 2010) (Supplementary Figure 5), supporting the idea that they act as endogenous *MYCN* regulators.
