# Supplementary material for: Tumour-suppressor microRNAs let-7 and mir-101 target the proto-oncogene MYCN and inhibit cell proliferation in MYCN-amplified neuroblastoma
Source: Br J Cancer. 2011 Jun 7;105(2):296–303. doi: 10.1038/bjc.2011.220 (PMC3142803; doi:10.1038/bjc.2011.220)

Supplementary figure 1

mir-34abc/449abc/699 seed family

Pos. 23-29

"wild-type"

3'UTR 5' ...UCAAAACUGGACAGUCACUGCCA...

|||||||

miRNA 3'...U**GUGACGG**U/A

"mut seed1"

5' ...UCAAAACUGGACAGUCACACCCA...

||| ||

3'...U**GUGACGG**U/A

5' ...UCAAAACUGGACAGUGTGACGGA...

"mut seed1 (7nt)"

Pos. 581-587

"wild-type"

3'UTR 5' ...CUGUACUAAUUCUUACACUGCCU...

|||||||

miRNA 3'...**GUGACGG**U/A

"mut seed2"

5' ...UCAAAACUGGACAGUCACACCCA...

||| ||

3'...**GUGACGG**U/A

mir-19 seed family

Pos. 32-38

"wild-type"

3'UTR 5' ...GACAGUCACUGCCAC--UUUGCACA...

|||||||

miRNA 3' ...CU**AAACGUG**U

"mut seed"

5' ...GACAGUCACUGCCAC--UUUCGACA...

||| ||

3' ...CU**AAACGUG**U

mir-29abc seed family

Pos. 334-340

"wild-type"

3'UTR 5' ...UCCUUCUUUUUAAAAUGGUGCUU...

|||||||

miRNA 3' ...U**ACCACGA**U

"mut seed"

5' ...UCCUUCUUUUUAAAAUGGACCUU...

||| ||

3' ...U**ACCACGA**U

mir-101 seed

Pos. 494-500

"wild-type"

3′UTR 5' ...GAAGUUCAUACCUAAGUACUGUA...
| | | | | | |
miRNA 3'...U**CAUGACA**U

"mut seed1"

5' ...GAAGUUCAUACCUAAGUAGAGUA...
| | | | |
3'...U**CAUGACA**U

Pos. 563-569

"wild-type"

3′UTR 5' ...GUUAAUCUCUGUUAU----GUACUGUA...
| | | | | | |
miRNA 3'...U**CAUGACA**U

"mut seed2"

5' ...GUUAAUCUCUGUUAU----GUAGAGUA...
| | | | |
3'...U**CAUGACA**U

mir-17-5p/20/93 seed family

Pos. 859-865

"wild-type"

3′UTR 5' ...GUGCUGCAUCUUAUAGCACUUUG...
| | | | | | |
miRNA 3'...**CGUGAAA**C

"mut seed"

5' ...GUGCUGCAUCUUAUAGCAGAUUG...
| | | | |
3'...**CGUGAAA**C

5' ...GUGCUGCAUCUUAUTCGTGAAAG...

"mut seed (7nt)"

Pos. 685-690 (Alternative seed according to MiRanda)

"wild-type"

3′UTR 5' ... CUUUAUCACUUUU...
| | | | | |
miRNA 3'...**CGUGAAA**C

"mut seed"*

5' ... CUUUAUCAGAUUU...
| | | |
3'...C**GUGAAA**C

let-7/98/202 seed family

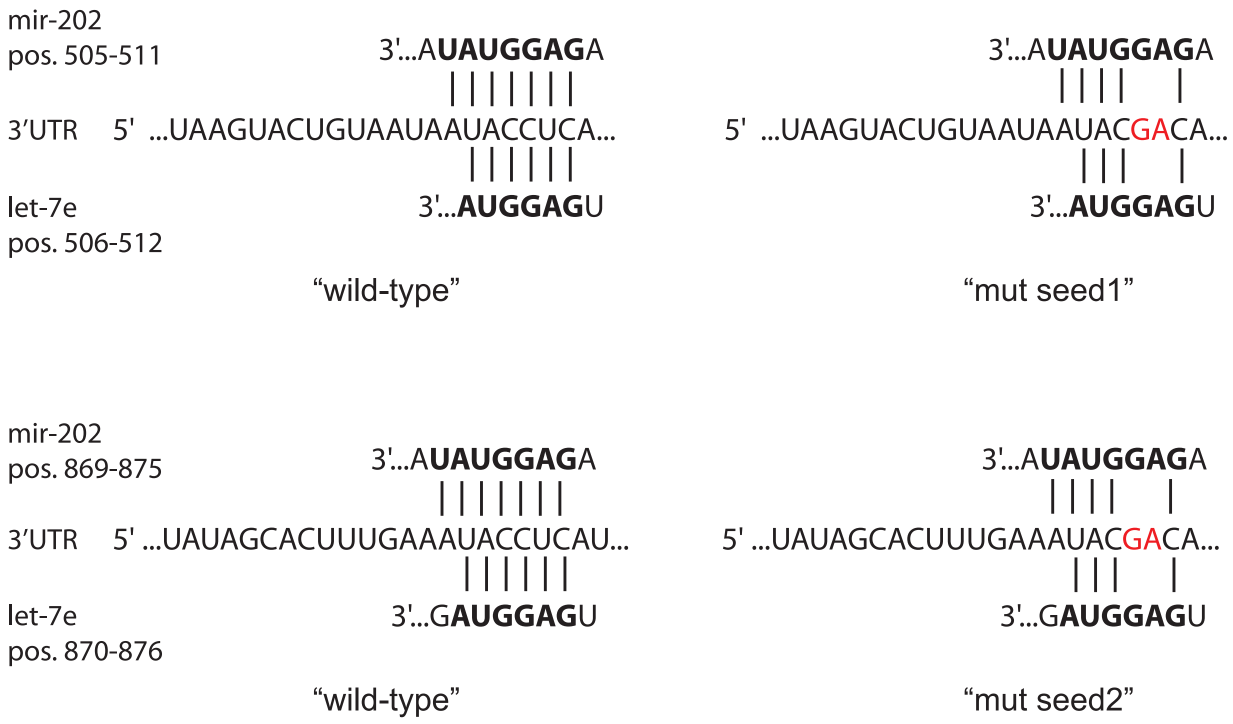

Supplement: Supplementary Figure 1 [file bjc2011220x1.pdf]
