# Supplementary figures and images for: Tumour-suppressor microRNAs let-7 and mir-101 target the proto-oncogene MYCN and inhibit cell proliferation in MYCN-amplified neuroblastoma
Source: Br J Cancer. 2011 Jun 7;105(2):296–303. doi: 10.1038/bjc.2011.220 (PMC3142803; doi:10.1038/bjc.2011.220)

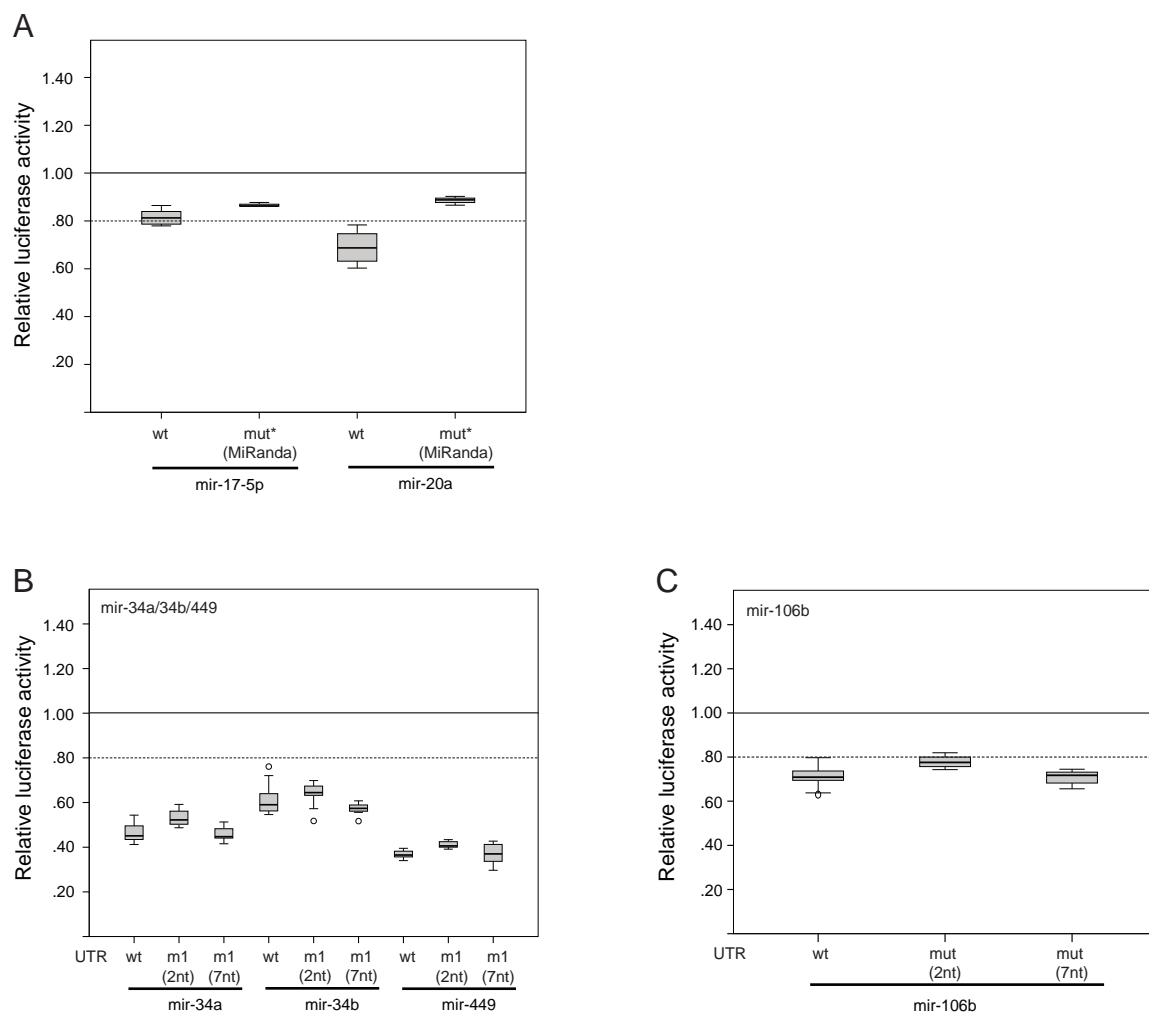

Supplementary Figure 2

Supplement: Supplementary Figure 2 [file bjc2011220x2.pdf]

A

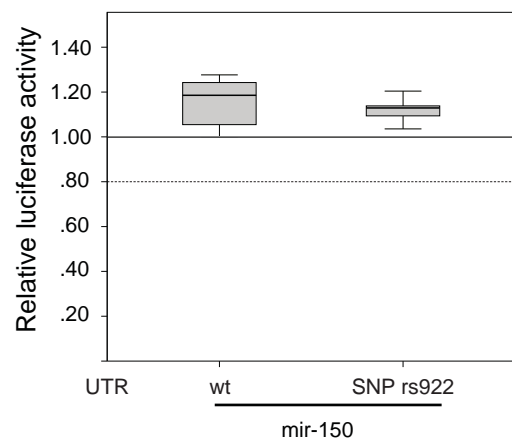

B

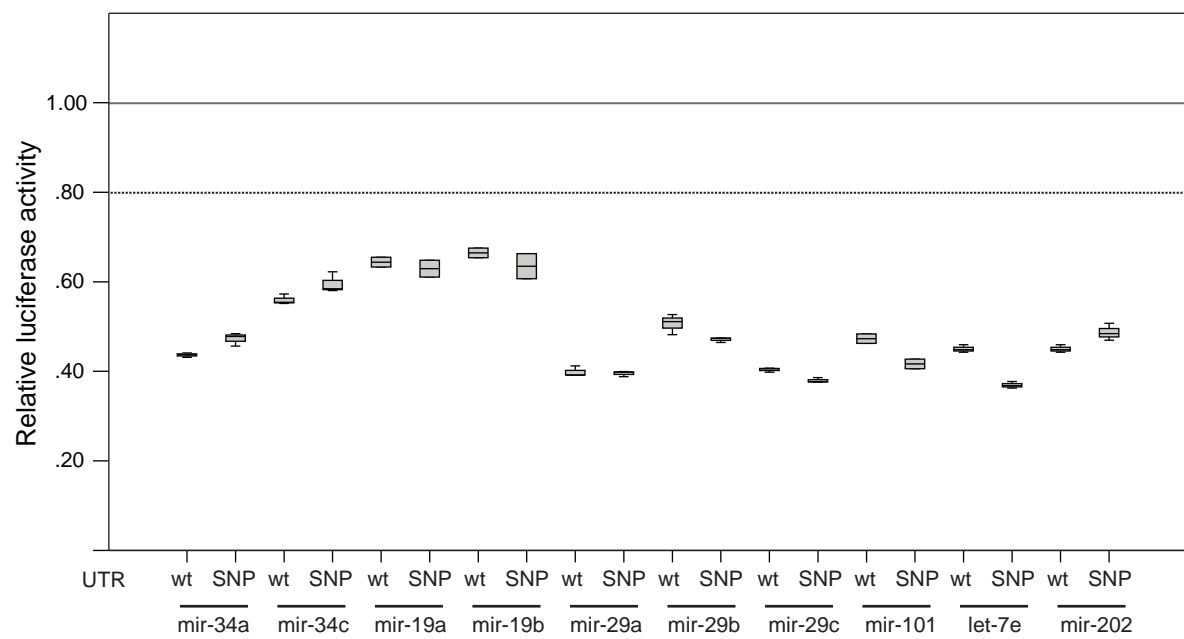

Supplement: Supplementary Figure 3 [file bjc2011220x3.pdf]

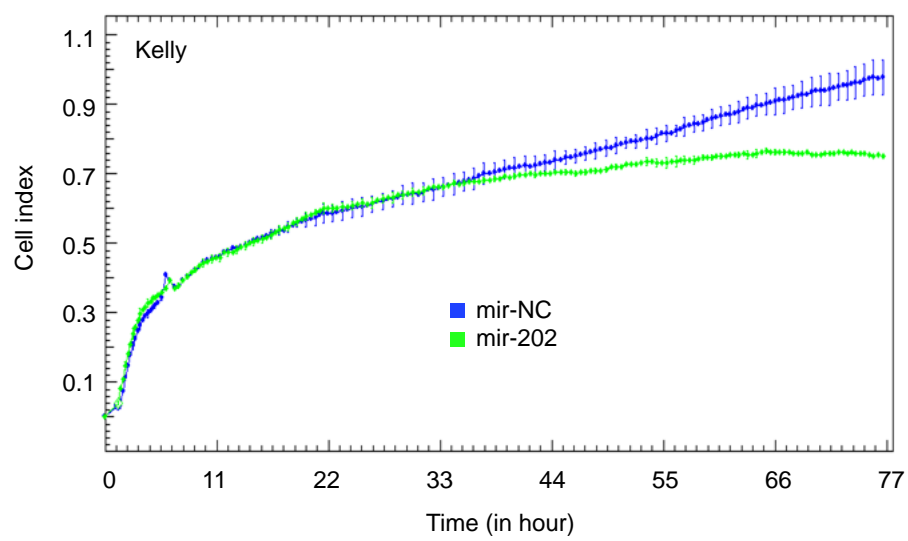

Supplementary Figure 4

Supplement: Supplementary Figure 4 [file bjc2011220x4.pdf]

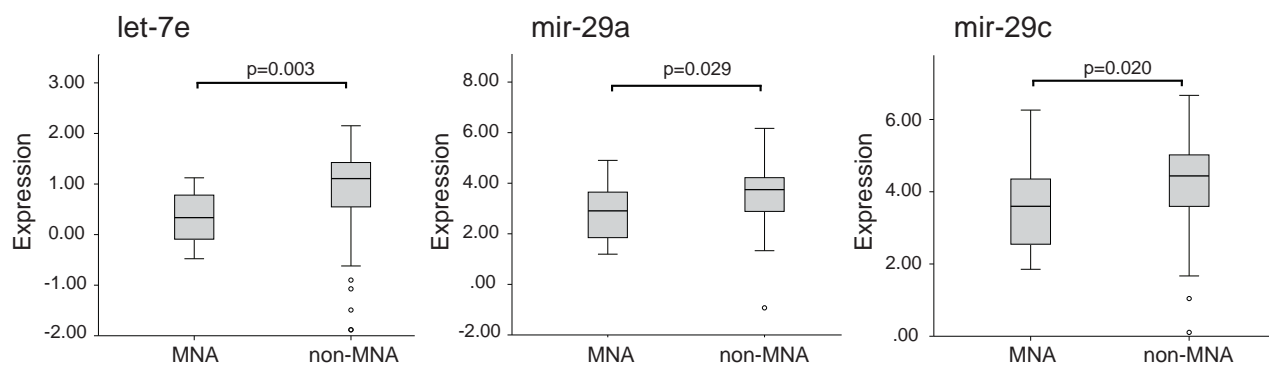

Supplementary Figure 5

Supplement: Supplementary Figure 5 [file bjc2011220x5.pdf]
