# Supplementary material for: Tumour-suppressor microRNAs let-7 and mir-101 target the proto-oncogene MYCN and inhibit cell proliferation in MYCN-amplified neuroblastoma
Source: Br J Cancer. 2011 Jun 7;105(2):296–303. doi: 10.1038/bjc.2011.220 (PMC3142803; doi:10.1038/bjc.2011.220)
Supplement: Supplementary Table 1 [file bjc2011220x6.pdf]

Supplementary table 1

Oligonucleotides used to clone miRNA expression vectors

pre-mir-34a	Forward	CAGTGGATCCAAGCTTGCCCTGGCCTCTCCAGTAGCTA
	Reverse	GTCTAGATATCTCGAGGGCCTTCCTGCATAGTAAGTGC
pre-mir-34c	Forward	CAGTGGATCCAAGCTTCTGTTAGCTCTCTCTAGGAGTC
	Reverse	GTCTAGATATCTCGAGTAAGAAAAAAATCATATTCCCC
pre-mir-106b	Forward	GATCAAGCTTCAGGCGTTACATAGCCATGT
	Reverse	GATCCTCGAGCTTGGGGAGCTCAGCCAAGA
pre-mir-150	Forward	CAGTGGATCCAAGCTTCTGGAGTCCACACTCCCTCTTT
	Reverse	GTCTAGATATCTCGAGGGGTTTTGCAGAGCTGGGGAGA
pre-mir-346	Forward	CAGTGGATCCAAGCTTCCCATGCCACTTGTCTTGCTTAA
	Reverse	GTCTAGATATCTCGAGCCCTTGGATGTGGGCCCCCCTT
pre-mir-449	Forward	CAGTGGATCCAAGCTTACATATTTTTATCCAAGCATTT
	Reverse	GTCTAGATATCTCGAGCAAAGACTCCAAAAATATTTCT

Oligonucleotides used to sequence expression vectors

pcDNA6.2EmGFP	Forward	GGCATGGACGAGCTGTACAA
	Reverse	CTCTAGATCAACCACTTTGT
pMiR-Report	Forward	GTTTTCCCAGTCACGACGTTGTA
	Reverse	ATCCTCATAAAGGCCAAGAA

Oligonucleotides used to clone of MYCN 3´UTR expression vector

Forward	ACGACCAAAAGATTGAACAAGATG
Reverse	GATCAAGCTTAATTTTAAGCTATTTATTTT

Oligonucleotides used to mutate specific seed sequences in pMIR-MYCN-UTR (2 nucleotides mutated)

Target site		miRNA	sequence
23-29	m1	mir-34a, miR-34c, miR-449	AACTGGACAGTCACACCCACTTTGCACATT
32-39	m1	miR-19a, miR-19b	GTCACTGCCACTTTCGACATTTTGATTTTT
334-340	m1	miR-29a, miR-29b, miR-29c	GTCATTCCTTCTTTTTAAAATGGACCTTAAGTTCCAGCAGATGCCAC
494-500	m1	miR-101	TTCATACCTAAGTAGAGTAATAATACCTC
506-512	m1	Let-7e, miR-202	CCTAAGTACTGTAATAATACGACAATGTTTGAGGAGCATG
563-569	m2	miR-101	ATCTCTGTTATGTAGAGTACTAATTCTTAC
581-587	m2	miR-34a, miR-34c, miR-449	ACTAATTCTTACACACCCTGTATACTTTAG
685-691		miR-20a, miR-17, miR-106b	GTTTTGAGTAGATATTACTTTATCAGATTTTGAACTAAGAAACTTTTG
859-865	m1	miR-106b, miR-17, miR-20a	TGCATCTTATAGCAGATTGAAATACCTCATG
870-876	m2	Let-7e, miR-202	GCATCTTATAGCACTTTGAAATACGACATGTTTATGAAAATAAATAGC

Oligonucleotides used to mutate specific seed sequences in pMIR-MYCN-UTR (7 nucleotides mutated)

Target site	miRNA	sequence
23-29	mir-34a, mir-34c, mir-449	CGCTTCTCAAAACTGGACAGTGTGACGGACTTTGCACATTTTGATTTTTTTTTAAAC
859-865	miR-106b	CTTCAAAATGTATATATTTAGTGCTGCATCTTATTCGTGAAAGAAATACCTCATGTTTATGAAAATAAATAGC

Oligonucleotide used to introduce the C250T mutation (SNP rs922) into pMIR-MYCN-UTR

Target site	sequence
250	CTAAACGTTGGTGATGGTTGGGAGCCTCTGG
