# Supplementary material for: Tumour-suppressor microRNAs let-7 and mir-101 target the proto-oncogene MYCN and inhibit cell proliferation in MYCN-amplified neuroblastoma
Source: Br J Cancer. 2011 Jun 7;105(2):296–303. doi: 10.1038/bjc.2011.220 (PMC3142803; doi:10.1038/bjc.2011.220)
Supplement: Supplementary Table 2 [file bjc2011220x7.pdf]

Supplementary table 2

miRNA seed family*	**screened miRNA**	MYCN UTR site position	/ site type	context score**	context score percentile
Conserved sites for miRNA families broadly conserved among vertebrates					
mir-101	**mir-101**	494-500	8mer	-0,43	95
		563-569	8mer	-0,46	97
mir-34abc/449abc/699	**mir-34a**	23-29	8mer	-0,41	98
		581-587	7mer-m8	-0,28	90
	**mir-34c**	23-29	8mer	-0,41	98
		581-587	7mer-m8	-0,31	93
	**mir-449ab**	23-29	8mer	-0,41	98
		581-587	7mer-m8	-0,32	94
let-7/98	**let-7e**	506-512	7mer-1A	-0,18	!#
		870-876	7mer-1A	-0,21	78
mir-19	**mir-19a**	32-38	8mer	-0,39	91
	**mir-19b**	32-38	8mer	-0,39	91
mir-144	**mir-144**	495-501	7mer-1A	-0,19	58
		564-570	7mer-1A	-0,17	50
mir-106/302	**mir-302e**	858-864	7mer-m8	-0,35	96
	**mir-372**	858-864	7mer-m8	-0,30	92
	**mir-373**	858-864	7mer-m8	-0,30	92
mir-17/20/93/106/519	**mir-17-5p**	859-865	7mer-m8	-0,35	93
	**mir-106b**	859-865	7mer-m8	-0,35	93
	**mir-93**	859-865	7mer-m8	-0,35	93
	**mir-20a**	859-865	7mer-m8	-0,35	93
mir-29abc	**mir-29a**	334-340	7mer-m8	-0,31	91
	**mir-29b**	334-340	7mer-m8	-0,31	90
	**mir-29c**	334-340	7mer-m8	-0,31	90
mir-200bc/429	**mir-200b**	466-472	8mer	-0,19	54
	**mir-429**	466-472	8mer	-0,19	55
Conserved sites for miRNA families conserved only among mammals					
mir-202	**mir-202**	505-511	8mer	-0,45	97
		869-875	8mer	-0,51	99
Poorly conserved sites and sites for poorly conserved miRNA families					
mir-150	**mir-150**	253-259	7mer-m8	-0,06	38

* Nomenclature according to TargetScan

** Grimson et al., Molecular Cell, Volume 27, Issue 1, 91-105, 6 July 2007
