# Supplementary material for: Tumour-suppressor microRNAs let-7 and mir-101 target the proto-oncogene MYCN and inhibit cell proliferation in MYCN-amplified neuroblastoma
Source: Br J Cancer. 2011 Jun 7;105(2):296–303. doi: 10.1038/bjc.2011.220 (PMC3142803; doi:10.1038/bjc.2011.220)
Supplement: Supplementary Figure and Table Legends [file bjc2011220x8.doc]

**Captions to supplementary materials**

**Supplementary Figure 1:**

**Putative *MYCN* 3’UTR / miRNA interactions predicted by TargetScan 5.1.** For each miRNA seed family, the wild-type (left) and mutant (right) seed sequences are shown. Mutations are shown in red.

**Supplementary Figure 2:**

**Luciferase reporter assays (LRAs) of *MYCN* 3’UTR / miRNA interactions. (A)** *Mir-17-5p* and *mir-20a* were investigated for binding to an alternative binding site uniquely predicted by the MiRanda software (Supplementary figure 1). Only a slight rescue in luciferase activity was observed for *mir-20a* combined with the mutated MiRanda-predicted target sequence (mut* MiRanda) when compared to the wt 3’UTR. Graphical presentations are similar to that described for Figure 2.

**Supplementary Figure 3:**

**The C to T variation at position 250 (SNP rs922) in the *MYCN* 3’UTR does not affect miRNA binding. (A)** LRAs showing that *mir-150* is neither able to suppress luciferase expression from the wild-type pMIR-MYCN-UTR vector (wt), nor the C250T-mutated version (SNP rs922). (B) LRAs showing that the C to T mutation at position 250 of the *MYCN* 3’UTR sequence did not substantially influence the suppressive activities of miRNAs validated to target *MYCN*.

**Supplementary Figure 4:**

72-hours continuous monitoring of cell proliferation after transfection of *mir-202* or a negative control (mir-NC) mimic into Kelly cells in 16-well E-plates on the xCELLigence system (Roche). After 35 hours, cell proliferation of the *mir-202* transfected cells was significantly inhibited when compared to the mir-NC control..

**Supplementary Figure 5:**

Box-plots of *let-7e*, *mir-29a* and *mir-29c* expression in 69 neuroblastomas (16 MNA and 53 non-MNA tumors), according to the expression data recently published by Schulte *et al*, (2010). The data is used here with permission of the authors. All three miRNAs were significantly lower expressed in MNA tumors compared to non-MNA tumors (p-values from Mann-Whitney U-tests).

**Supplementary Table 1:**

Oligonucleotides used in this study

**Supplementary Table 2**: Panel of predicted *MYCN*-targeting miRNAs investigated in our study by luciferase reporter assays. Twenty broadly conserved miRNAs were selected to cover 13 conserved miRNA target sites in the *MYCN* 3'UTR. *Mir-202*, a miRNA conserved only among mammals with two conserved binding sites was included. *Mir-150* is a non-conserved miRNA with a predicted target site in proximity to the rs922 SNP. Context scores and context score percentiles are according to TargetScan .
